# French agroclimatic research network dataset

**DOI:** 10.1016/j.dib.2025.111809

**Published:** 2025-06-20

**Authors:** Carina Furusho-Percot, Olivier Maury, Vincent Minet, Timothée De Decker, Daniel Roux, Claire Beauvois, Renan Le Roux, Jérémie Décome, Marie Launay, Iñaki García de Cortázar-Atauri

**Affiliations:** INRAE, AgroClim, Avignon, 84140, France

**Keywords:** Climate, Weather, Temperature, Precipitation, Wind, Radiation, Wetness, Humidity

## Abstract

The AgroClim dataset includes multi-decade long time-series of daily and hourly data from weather stations composed by the same twelve instruments at 54 sites throughout the French territory, including Corsica, Guyana and Guadeloupe. A large diversity of INRAE research projects benefited from the reliable climatological data from this dataset for decades. Now that the dataset is provided in open access for all, this paper describes the acquisition methods and maintenance that ensure the quality of the data. This data can contribute to enlarge observatories and observation datasets or assimilated, for producing reanalysis dataset, and ultimately used for validation of earth system simulations. Furthermore, this dataset contains rarely available measures as leaf wetness duration and soil temperature at 10 and 50 cm depths, which are useful for crop models and carbon, nitrogen and phosphorus cycle modelling. Moreover, the sites are located on research experimental sites, which contain data from long term agronomy experiments and can contribute to the study of a variety of bio-climatological studies.

Specifications Table

**Table:** 

Subject	Earth & Environmental Sciences
Specific subject area	Local climatic measurements (air, soil temperature, precipitation, air humidity, wind, solar radiation, leaf wetness) on agricultural research sites.
Type of data	Table CSV, Raw, Filtered.
Data collection	Hourly and daily climatic data are collected from 54 stations located in research fields in France, including Corsica, Guadeloupe and Guyana. Each station is equally managed and maintained by AgroClim INRAE (https://agroclim.inrae.fr/), composed by a central acquisition system powered by a solar panel and the same 12 sensors: five thermometers, a tipping rain gauge, a humidity probe, a pyranometer, an optical filter, a wetness sensor, an anemometer and a digital windvane. They measure air temperature under shelter, air humidity under shelter, wind speed and direction, precipitation, global solar radiation, photosynthetically active radiation, leaf wetness duration, air-temperature at 10 and 50 cm, and soil temperature at 10 and 50 cm depths. The daily data is filtered to remove main outliers, with season-dependent thresholds. Hourly data is raw and not yet filtered nor validated.
Data source location	The measurement locations (latitude, longitude and altitude) are listed at https://agroclim.inrae.fr/carto/, in Mainland France, Corsica, Guyana and Guadeloupe. The data is managed by AgroClim at INRAE in Avignon (https://agroclim.inrae.fr/) and stored at the facilities of INRAE Toulouse, France.
Data accessibility	Repository name: https://entrepot.recherche.data.gouv.fr/dataverse/agroclim Data identification number: DOI:10.57745/BQ0BWB Direct URL to data: 10.57745/BQ0BWB
Related research article	Vidal, Tiphaine, Anne-Lise Boixel, Essia Maghrebi, Rémi Perronne, Philippe du Cheyron, Jérôme Enjalbert, Marc Leconte, et Claude de Vallavieille-Pope. 2022. « Success and Failure of Invasive Races of Plant Pathogens: The Case of Puccinia Striiformis f. Sp. Tritici in France ». *Plant Pathology* 71 (7): 1525‑36. https://doi.org/10.1111/ppa.13581. [1]

## 1 Value of the Data

• Long time-series of climatic measured data can be used to study the impact of climate on agricultural systems, as for example the effect of warm spells, cold spells, droughts and lack of solar radiation on crops.
• This dataset can contribute with quality data for larger observatories (as FAOCLIM) and observation dataset, assimilated for producing reanalysis dataset, and ultimately used for validation of earth system simulations.
• This dataset includes one of the largest network of leaf wetness duration measurements, of great importance for the study of plant diseases, measured with the same type of sensor in diverse climatic regions.
• The measurement stations are located on research experimental sites, which contains data from long term agronomy experiments and can contribute for the study of a variety of bio-climatological studies and comparison of cropping systems resilience. Moreover, these data can be used as input to crop models and plant disease models.
• The INRAE network is partner of Météo-France, thus values of classical meteorological variables are transferred to Météo-France for weather forecast and data aggregation.
• A large diversity of research projects benefited already from the reliable climatological data from this data collection. Examples include research in soil carbon stock in agroforestry [2], cropping practices [3], insect invasion [4], plant diseases [1,5] and many others (https://agroclim.inrae.fr/climatik/help/citation/publications-associees/)

## 2 Background

Climate drives and affects agricultural systems in several ways. AgroClim measurement network was created to provide reliable data on various variables of interest for agricultural experimentation and research throughout the French territory, including Corsica, Guyana and Guadeloupe.

This data contributes to quality data check for observation datasets, assimilated for producing reanalysis dataset, and evaluation of climate and earth system models [[6], [7], [8]]. Moreover, this dataset contains rarely available measures as leaf wetness duration and soil temperature at 10 and 50 cm depths, which are useful for plant disease studies, for biochemical and hydrological processes for carbon, nitrogen and phosphorus cycle modelling. For example, soil extreme hot temperatures outpace air temperature twice as fast over Central Europe and “maximum soil temperatures should be included in impact and risk studies as a complementary perspective to the traditional approach.” [9].

This dataset contributed to a variety of bio-climatological studies for researchers and partners of INRAE. This data collection is now openly available as part of INRAE’s commitment to open science. We believe that sharing research data not only with the scientific community but also with society at large advances knowledge and builds trust-based relationships with a diverse range of stakeholders.

## 3 Data Description

### 3.1 Final Data Format and File Structure

**Fig. 1:**
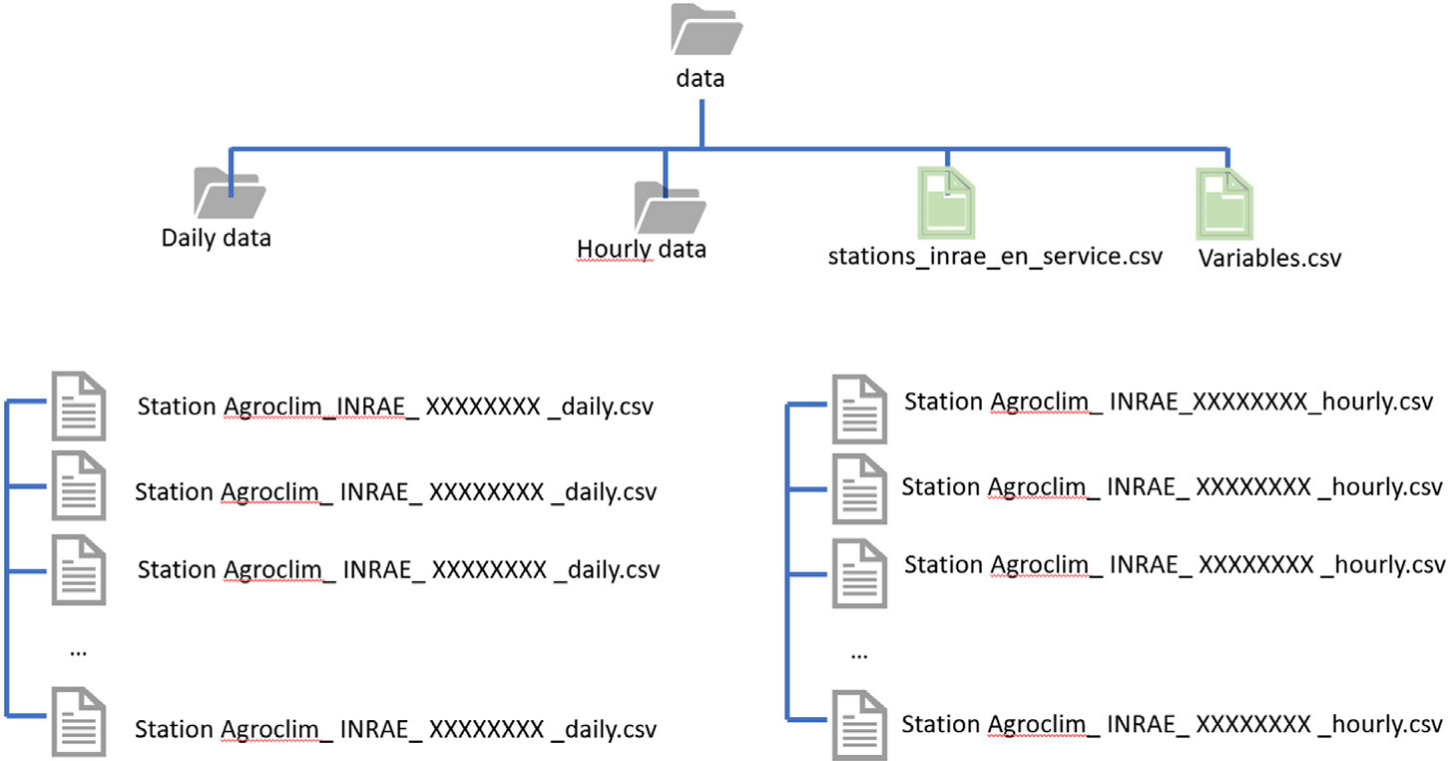
Data files structure.

**Fig. 2:**
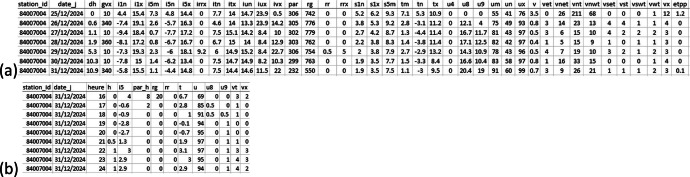
Example of the data content, illustrated by a fragment of time-series for the weather station located in Avignon (84007004) in daily (a) and hourly (b) time-step.

The data [10] is divided into two groups: one for daily and the second for hourly data. The groups can be filtered by the tags “daily” and “hourly” in the data repository^1^ (represented by two folders in Fig. 1). Each group is composed by 54 CSV format files, where each column name corresponds to the measured variable (Fig. 2).

Three files are tagged as “Documentation” in the dataset repository:

• “README.md”,
• “Variables.csv”, and
• “stations_inrae_en_service.csv”.

The 11 hourly and 40 daily data are listed in the file “Variables.csv”, presenting each variable’s name, unit and description in French and in English.

The list of 54 weather stations is provided in the file “stations_inrae_en_service.csv”, containing the geographical location with the name of the city, latitude, longitude and altitude (Table 1). It also provides the date the station started recording the data and the date of any dislocation of the station within the parcel to avoid the proximity of new buildings or trees that could hinder the measures of solar radiation, precipitation or wind.

**Table 1:** Extract from the list of the current 54 stations in the file “stations_inrae_en_service.csv”.

ID	City	Location	Altitude (m)	Lon (°Dec)	Lat (°Dec)	Start date	Relocation date
11170004	GRUISSAN	PECH-ROUGE	40	3,13551	43,14322	01/11/1989	17/06/1994
12232002	SAINT-JEAN	LA FAGE	700	3,09523	43,91937	16/11/1988	10/05/1996
17353002	SAINT-LAURENT	MECHE	3	-1,02732	45,98846	01/08/1985	06/10/1994
33550003	VILLENAVE-D'ORNON	LA GRANDE FERRADE	25	-0,57649	44,78998	01/09/1991	-
61328003	LE PIN-AU-HARAS	DOMAINE DE BORCULO	204	0,18094	48,72507	25/02/2010	-

**Fig. 3:**
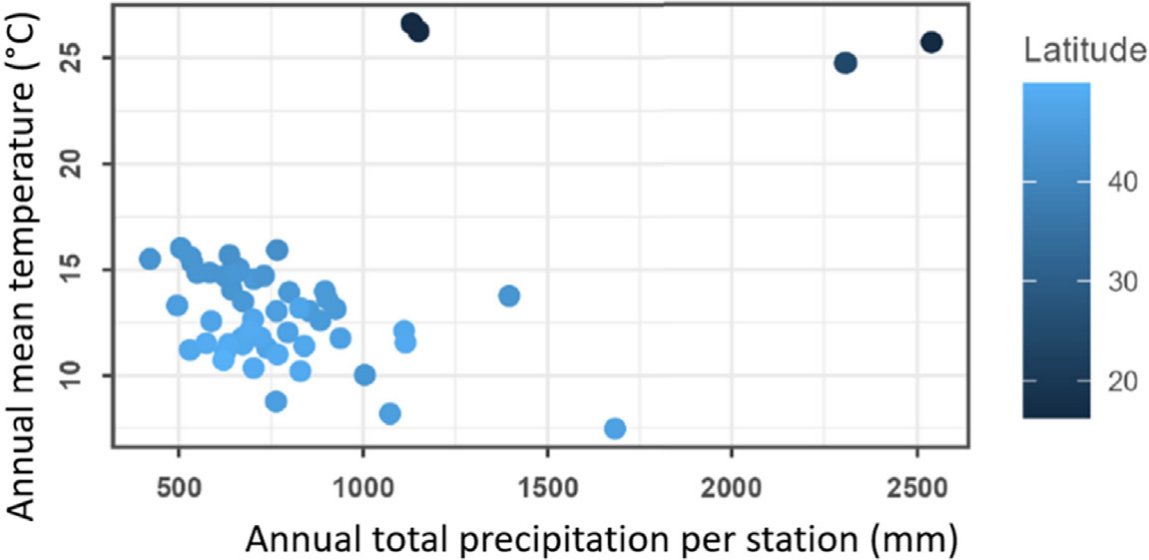
Annual mean temperature and precipitation for each one of the 54 INRAE agroclimatic stations of this dataset, extracted from station summary charts [11]. The four stations located in the tropics (Guyana and Guadeloupe) are represented by the darker dots (lower latitudes), with the highest mean annual temperatures and the higher total annual precipitation in the network.

The stations cover a variety of climatic conditions, with total precipitation ranging from 200 to 2500 mm per year and mean annual temperature between 7 and 27°C (Fig. 3). An ensemble of 54 charts summarizes local weather and climate conditions through a series of graphs and tables [11].

## 4 Experimental Design, Materials and Methods

**Fig. 4:**
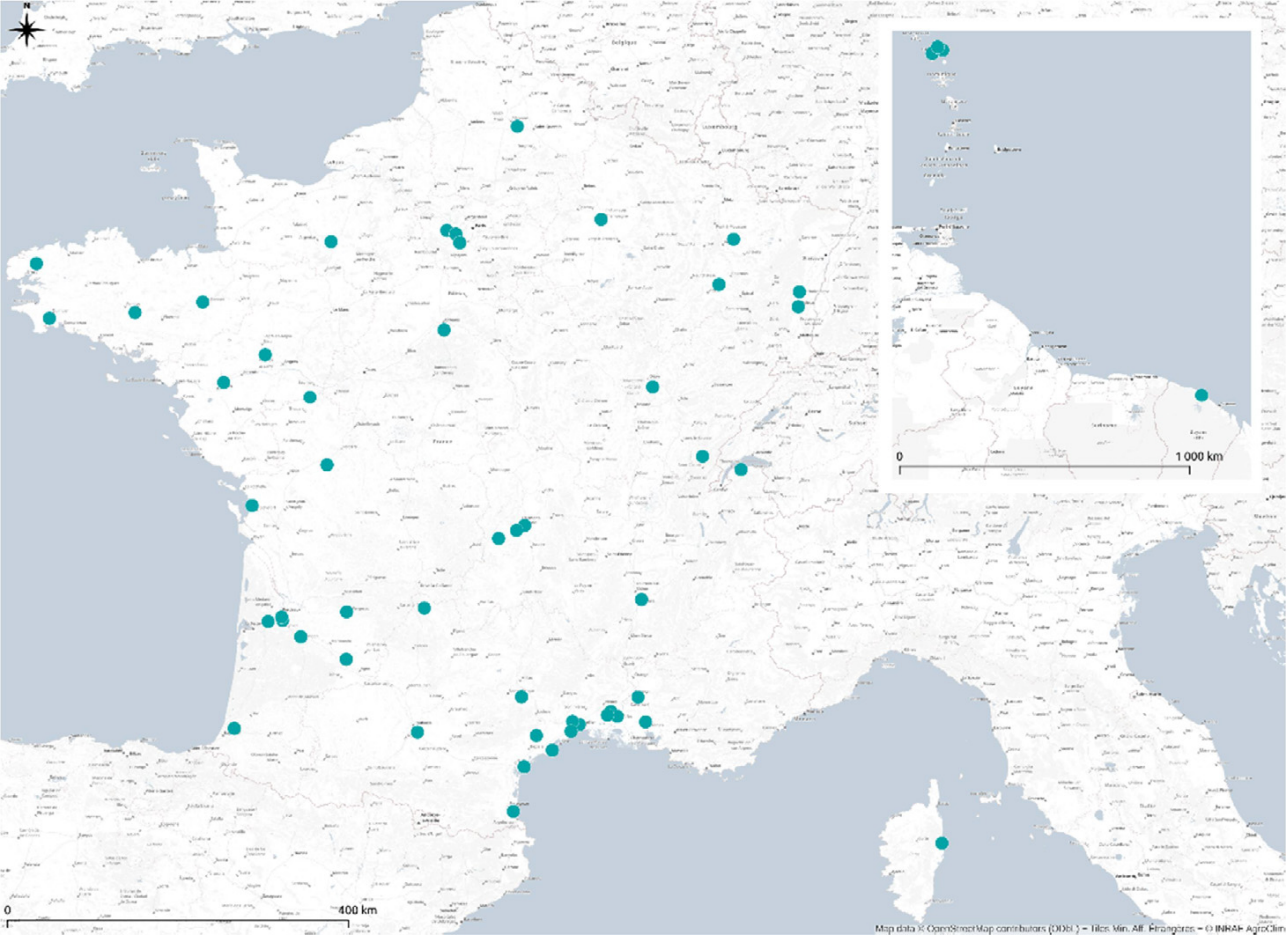
Map of stations of the INRAE national agroclimatic network currently running.

**Fig. 5:**
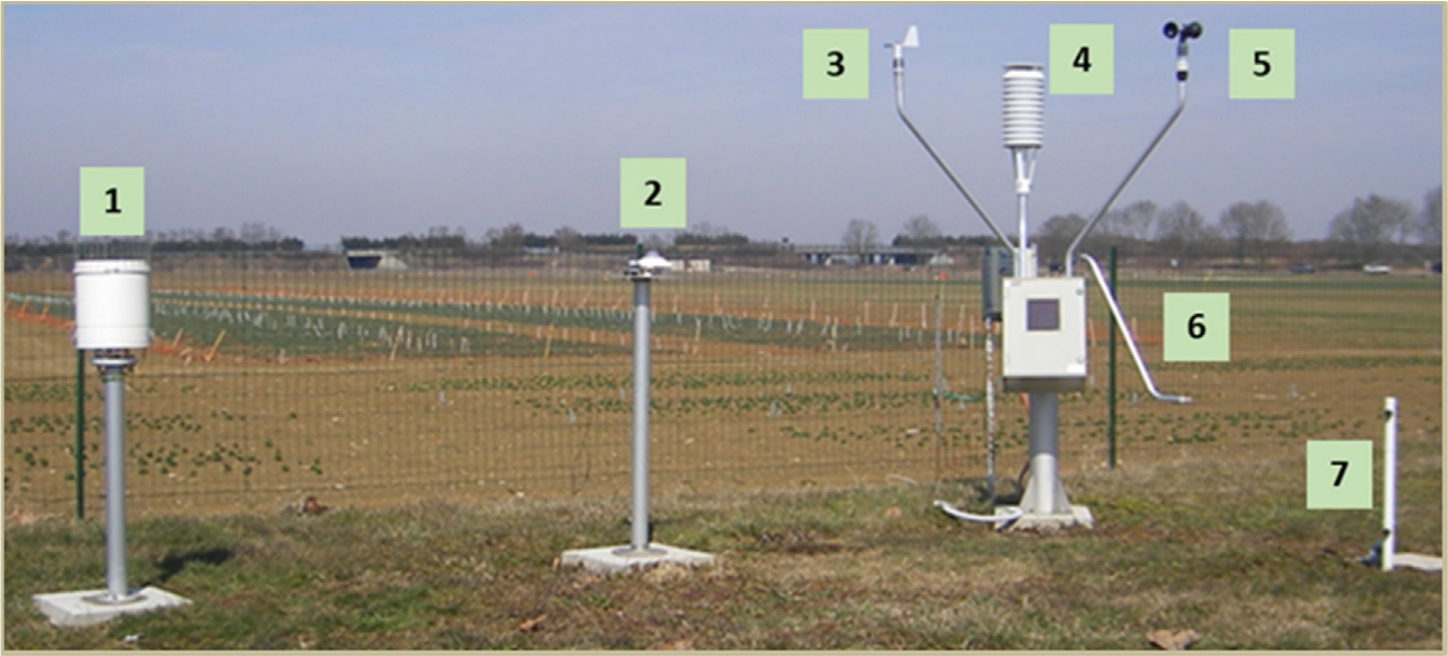
Standard INRAE national agroclimatic network station. (1) rain gauge; (2) global radiation and PAR measurement; (3) wind direction measurement; (4) air temperature (2m) and humidity measurement; (5) wind speed measurement; (6) leaf wetness duration sensor; (7) temperatures at +10 and +50cm above the soil surface) and soil temperature measurement (-10 and -50cm). The system is autonomous thanks to the solar panel and the battery placed at the central acquisition box under the temperature shelter (4) and the data is transmitted by General Packet Radio Service (GPRS).

The INRAE's agroclimatic network currently comprises 54 weather stations in mainland France, French Guyana and Guadeloupe [12]. 47 stations are located at INRAE and 7 at partner experimental sites (Fig. 4). The stations are all equipped with the same hardware, i.e sensors, acquisition system and infrastructure (Fig. 5). A weekly station monitoring is performed by local staff at each site. In addition, AgroClim performs an annual complete site inspection, calibration and monitoring of equipment. Local maintenance operations are tracked via a web application, enabling us to check that the network is running smoothly.

Our agroclimatic network measures climatic variables with the sensors (Table 2) as displayed in Fig. 5: (4) air temperature under shelter, (4) air humidity under shelter, (5) wind speed, (3) wind direction, (1) rain and (2) Global Solar Radiation (GSR), at hourly intervals. The measurement conditions are standardized at the reference height of 2 m for measurements (3, 4, and 5) and 1.5 m for measurements (1, 2). These standard measurements are accompanied by specialised measurements which include (6) leaf wetness duration at 50 cm, (2) Photosynthetically Active Radiation (PAR) and (7) temperature measurements 10 and 50 cm above and deep the soil (7). The data are retrieved from the stations to a concentrator every 30 min from 6:00 to 21:00 (The rhythm and concentration time range are adjustable throughout the year depending on battery condition). The data concentration is performed through cimnet application (from cimel company) on a server located in the INRAE data center in Toulouse. The data is then transferred to a database for filtering out suspicious data. The data is then transferred to the Climatik application database in real time (restricted to INRAE agents) and we intend to update this open collection once a year.

**Table 2:** Description of current instruments, stable and homogeneous since 2007.

MEASURE	INSTRUMENT	TYPE	MAINTENANCE
CENTRAL ACQUISITION	Acquisition	GPRS Cimel 516i	Weekly check
WIND SPEED	Cup-shaped anemometer	Cimel CE 155 N	Weekly check
WIND DIRECTION	Digital windvane	Cimel CE 157 N	Weekly check
LEAF WETNESS DURATION	Leaf wetness sensor	Cimel HU 1871	Weekly check, bi-annual replacement
HUMIDITY	humidity transmitter probe	Vaisala HMP110	Weekly check, annual calibration
PHOTOSYNTHETICALLY ACTIVE RADIATION	Optical Filter	PAR Lite Kipp&Zonen	Weekly check, annual calibration
GLOBAL RADIATION	Pyranometer	CMP6 Kipp & Zonen	Weekly check, annual calibration
PRECIPITATION	Tipping raingauge 0.5mm	Précis mécanique RS53029	Weekly check And annual calibration
2M/50CM/10CM AIR TEMPERATURE	PT 100 4 fils	JD Mesure	Weekly check
10 AND 50 CM DEPTH SOIL TEMPERATURE	PT 100 4 fils	JD Mesure	Weekly check

The detailed on-site maintenance is performed annually in mainland France, and every 2 years in Corsica, Guadeloupe and French Guyana. Preventive maintenance operations are carried out in the presence of the station's local technical contact. All sensors and measurement channels are checked, some are replaced for calibration (hygrometry and radiation sensors), and faulty parts are repaired or replaced (sensor connectors, chimney shelters, bearings, etc.). Monitoring the rain gauge is the most time-consuming operation in all maintenance and consists in the following procedure:

- Check the serial number.
- Switch to test mode in the data acquisition central.
- Clean the entire rain gauge with clean water.
- Check that the water is draining properly. Clean the drain filter if necessary.
- Check that the support is secure and tighten it if necessary.
- Check that the tipping buckets are working correctly by placing a level (such as a ruler) on both sides.
- Weigh the buckets with a syringe and adjust if necessary, using the screws.
- Check the connections (capacitor and lugs).
- Run a rain test with 1 litre of water (measured with a test tube).

Expected result: 48 to 51 tilts (0.5 mm buckets).

If the result is not correct: adjust the buckets. Weight them before a new rain test.

- Stop Test mode. Reminder: it deactivates automatically after 2 h otherwise.
- Replace the rain gauge only if it does not comply (because it cannot be adjusted).

For the other sensors, the procedure consists on verifying the serial number, clean the support and the probes, check the connections and the instantaneous value. Finally, the technician makes a call from the station to send the data and check that the hourly values have been received on CimNet concentrator.

Our calibration laboratory is composed by the equipment (Table 3) and comprises 3 stations:

- A global radiation calibration bench consisting of a 1m^3^ black box, equipped with a 1000 W halogen lamp and a ventilation system to control temperature rise in the box. The bench is set for comparing the instruments to the reference pyranometer.
- A relative humidity calibration bench. A Rotronic Hydrogen-type humid air generator supplies relative humidity and temperature. In this environment, our sensors are compared to the Hygroclip standard reference.
- A temperature calibration bench consisting of an ISOTECH Hydra 98M thermostatic bath, where the fluid temperature varies from -20 to 60°C. The reference temperature probe is immersed in this liquid along with the probes to be calibrated.

**Table 3:** Description of calibration laboratory equipment.

EQUIPMENT	BRAND	TYPE	CALIBRATION FREQUENCE
GLOBAL RADIATION	Kipp & Zonen	CM 21	Every 2 years
HUMIDITY GENERATOR	Rotronic	Hygroclip	Controlled every 5 years
CONDENSATION HYGROMETER	Michell instrument		Annual
DRY BULB TEMPERATURE	Michell instrument		Annual
REFERENCE TEMPERATURE STANDARD	AOIP	PHP 602	Annual
THERMOSTATIC BATH	AOIP	AN 5847	Controlled every 5 years
PAR LI-COR	LI-COR	LI-190	Annual
ELECTRICAL MEASUREMENTS	AOIP	JN 5305	Annual

We monitor the changes in the weather station site environment to comply with Météo-France’s requirements, as the minimum distance of 20 m from any building, tree or irrigation that could influence the measurements. Following this visit, an inspection and maintenance certificate is delivered to the experimental site unit manager and the site's technical contact. Finally, every 3 - 4 years, the AgroClim unit organizes on-site (in Avignon) training days for the network's technical contacts. These meetings provide a technical and methodological reminder of the control steps to be carried out each week on the station, and to present the unit's activity and the use of network data. These meetings also provide an opportunity to foster the technical network and share good practices.

### 4.1 Daily Data Filter

We applied filters to detect outliers in daily data based on robust and efficient estimators of the mean and standard deviation (biweight) [13]. The main variables were filtered based on their statistic distribution and related variables (that depend on the measures made by the same sensor on that day) were considered as non-valid as follows (see “Variables.csv” for the meaning of variable abbreviations):

• The components of daily wind from all directions (VET, VNET, VNT, VNWT, VSET, VST, VSWT, VWT) are excluded, if the total wind measure (V) is not valid, because any of the components of the wind vector may be false if the total measure is false;
• Time of occurrence of the maximum intensity of rainfall, temperature and moisture (IRRX, ITN, ITX, IUN, IUX, IVX) are excluded when the corresponding measures are not valid;
• Cardinal direction of the wind with the maximum daily intensity (GVX) is excluded if the maximum wind intensity (VX) is not valid;
• Maximum daily precipitation (RRX) is excluded if daily precipitation (RR) is not valid, because if the total amount of precipitation is false, the value that was measured as the maximum intensity is likely also false;
• Humidity lower than 40 % (U4), higher than 80 % (U8), and higher than 90 % (U9) are excluded if the average daily humidity (UM) is not valid.

## Limitations

This set of raw data requires further validation and gap-filling before feeding statistical trend analysis and crop models, even if daily data has been automatically filtered to remove outliers [13]. Hardware changes (as sensor replacement) and lack of measurement consistency due to sensor malfunctioning (as clogged raing gauge or dirty pyranometer until the weekly intervention) may have introduced undetected artificial biases and errors in the time series, which can impact the comparability over time.

## Ethics Statement

The authors have read and follow the ethical requirements for publication in Data in Brief and confirm that the current work does not involve human subjects, animal experiments, or any data collected from social media platforms.

## CRediT authorship contribution statement

**Carina Furusho-Percot:** Writing – original draft, Writing – review & editing. **Olivier Maury:** Software, Writing – review & editing. **Vincent Minet:** Software, Writing – review & editing, Data curation. **Timothée De Decker:** Software, Writing – review & editing, Data curation. **Daniel Roux:** Investigation, Supervision, Writing – review & editing. **Claire Beauvois:** Investigation, Writing – review & editing. **Renan Le Roux:** Software, Writing – review & editing. **Jérémie Décome:** Software, Writing – review & editing. **Marie Launay:** Project administration, Writing – review & editing. **Iñaki García de Cortázar-Atauri:** Project administration, Writing – review & editing.

## Data Availability

DataverseDaily and hourly climatic data from 54 stations of the INRAE Agroclim network (Original data).

## Footnotes

1 https://doi.org/10.57745/BQ0BWB.
